# Mitigating antimicrobial resistance through effective hospital wastewater management in low- and middle-income countries

**DOI:** 10.3389/fpubh.2024.1525873

**Published:** 2025-01-29

**Authors:** Yaovi Mahuton Gildas Hounmanou, Adonias Houefonde, I-CRECT Consortium, Linh Viet Nguyen, Tram Thuy Nguyen, Anders Dalsgaard

**Affiliations:** ^1^Department of Veterinary and Animal Sciences, Faculty of Health and Medical Sciences, University of Copenhagen, Frederiksberg, Denmark; ^2^Polytechnic School of Abomey-Calavi, University of Abomey-Calavi, Abomey-Calavi, Benin; ^3^The National Institute of Hygiene and Epidemiology (NIHE), Hanoi, Vietnam

**Keywords:** hospital wastewater, antimicrobial resistance, antibiotic-resistant genes, antibioticresistant bacteria, hospital wastewater management

## Abstract

Hospital wastewater (HWW) is a significant environmental and public health threat, containing high levels of pollutants such as antibiotic-resistant bacteria (ARB), antibiotic-resistant genes (ARGs), antibiotics, disinfectants, and heavy metals. This threat is of particular concern in low- and middle-income countries (LMICs), where untreated effluents are often used for irrigating vegetables crops, leading to direct and indirect human exposure. Despite being a potential hotspot for the spread of antimicrobial resistance (AMR), existing HWW treatment systems in LMICs primarily target conventional pollutants and lack effective standards for monitoring the removal of ARB and ARGs. Consequently, untreated or inadequately treated HWW continues to disseminate ARB and ARGs, exacerbating the risk of AMR proliferation. Addressing this requires targeted interventions, including cost-effective treatment solutions, robust AMR monitoring protocols, and policy-driven strategies tailored to LMICs. This perspective calls for a paradigm shift in HWW management in LMIC, emphasizing the broader implementation of onsite treatment systems, which are currently rare. Key recommendations include developing affordable and contextually adaptable technologies for eliminating ARB and ARGs and enforcing local regulations for AMR monitoring and control in wastewater. Addressing these challenges is essential for protecting public health, preventing the environmental spread of resistance, and contributing to a global effort to preserve the efficacy of antibiotics. Recommendations include integrating scalable onsite technologies, leveraging local knowledge, and implementing comprehensive AMR-focused regulatory frameworks.

## 1 Introduction

Hospitals play a vital role in national healthcare systems and advancing medical science (1). However, their operations generate wastewater containing diverse hazardous contaminants, posing major challenges for environmental health. Hospital wastewater (HWW) is rich in pollutants, including toxic chemicals, antibiotic residues, pathogens, and antibiotic-resistant bacteria (ARB) and genes (ARGs) (2, 3). Importantly, contaminant levels in hospital wastewater are considerably higher that those in community wastewater (4, 5), making hospitals critical hotspots for antimicrobial resistance (AMR) and environmental pollution.

Updated global estimates indicate that, in 2021, 1.14 million deaths were directly attributable to bacterial antimicrobial resistance (AMR), with most occurring in low- and middle-income countries (LMICs) due to diverse and complex factors (6). However, the environmental contribution to this burden remains largely overlooked, despite its potential role as a key driver (7). Currently, there are no precise figures quantifying the environmental contribution to the overall AMR burden. While the clinical implications of environmental antibiotic resistance remain poorly understood, environmental bacteria, among the most abundant microbial populations, can serve as reservoirs and donors for resistance genes. Through horizontal gene transfer, these genes may ultimately reach human and animal pathogens (8). Moreover, disinfectants, heavy metals, and antibiotic residues in hospital wastewater (HWW) may act as co-selectors for AMR, compounding the problem (2).

In LMICs, untreated HWW poses significant risks to public health and the environment due to insufficient onsite treatment infrastructure. Most hospitals in LMICs discharge wastewater without proper treatment, leading to the widespread dissemination of ARGs, ARB, and other hazardous substances (9–11). Untreated HWW is often used for irrigating crops, especially leafy crops, increasing direct human exposure to ARGs, ARB, and toxic substances through food consumption (12). Vulnerable populations living downstream HWW discharge points face elevated risk from direct exposure to untreated wastewater in these regions (13, 14). This practice exacerbates the environmental dissemination of ARGs, posing risks to food safety and public health through direct exposure to resistant pathogens.

This perspective advocates for a paradigm shift in HWW management in LMICs, emphasizing the need for onsite treatment systems that target AMR alongside traditional pollutants. Key steps include developing cost-effective technologies specifically designed to eliminate ARGs and ARB, implementing AMR-focused monitoring standards, and protecting vulnerable populations from exposure. Addressing these challenges is vital not only for public health in LMICs but also for the global effort to preserve antibiotic efficacy.

## 2 Treatment of hospital wastewater in LMICs: effectiveness and perspectives for addressing AMR

**Figure 1:**
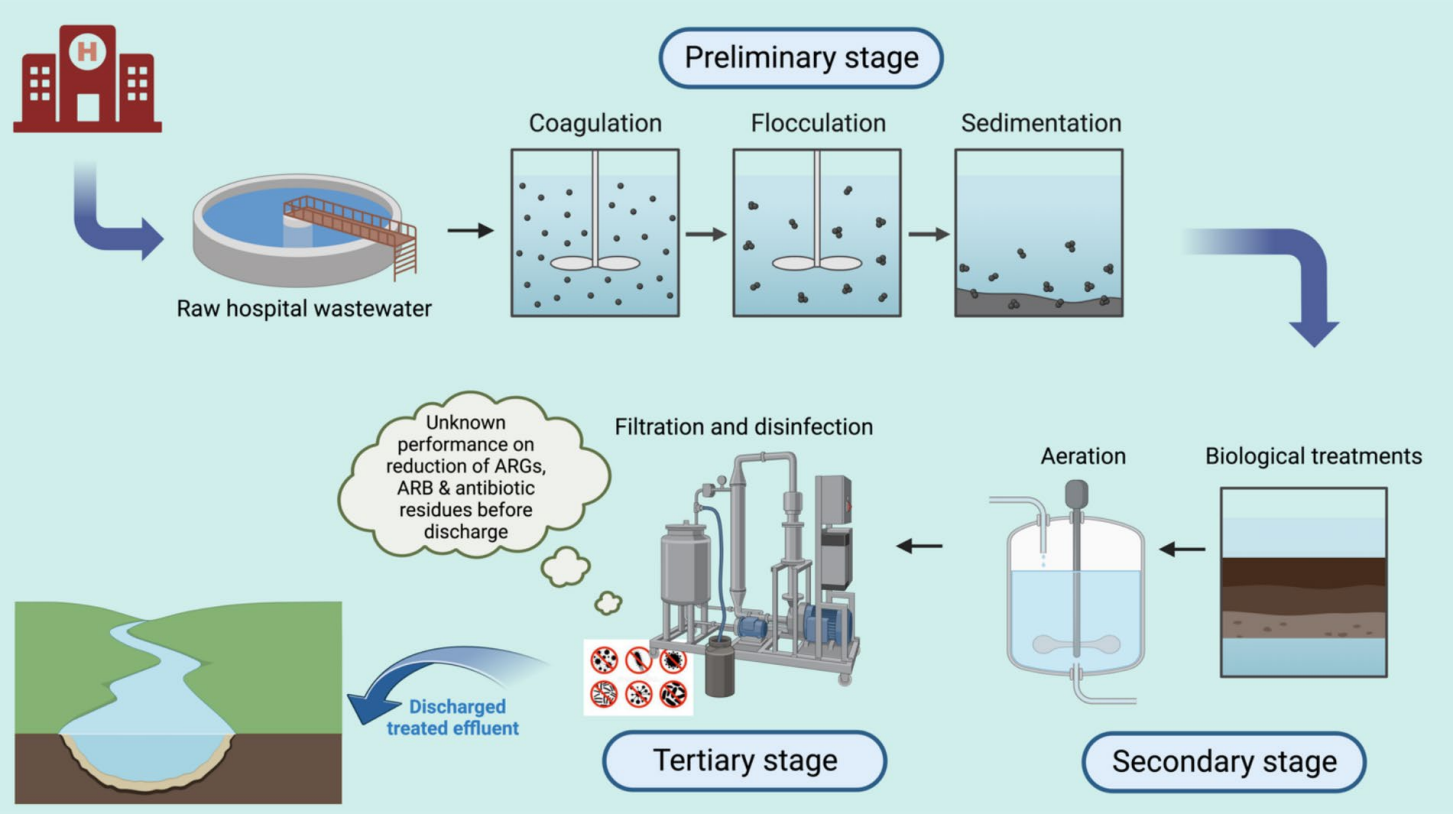
Representation of typical HWWT processes described in the existing literature from LMICs. Created in Biorender.com.

Onsite treatment of hospital wastewater is uncommon in many LMICs, where most hospitals discharge untreated wastewater directly into the environment. This practice poses considerable environmental and public health risks, particularly in the spread of AMR. In LMICs where onsite treatment is implemented, it typically involves a combination of physical, chemical, and biological processes aimed at removing solids and organic matter through three main stages (Figure 1) (15, 16). The first stage is primary sedimentation, followed by secondary treatment with aerobic biological processes such as activated sludge, trickling filters, or rotating biological contactors. The final, tertiary stage involves disinfection. However, these systems often prioritize the removal of conventional pollutants and are generally insufficient for effectively eliminating ARB, ARGs, and antibiotic residues. Inconsistent disinfection practices, along with issues such as high sludge production, formation of potentially toxic by-products, and limited monitoring for AMR components, further reduce the efficacy of these systems.

These primary and secondary treatment stages may reduce organic loads but do little to address AMR, while tertiary disinfection methods are inconsistently applied. Key limitations to these approaches include:

- First, the inexistence of any on-site wastewater treatment system in majority of LMICs hospitals.
- Lack of infrastructure for advanced treatment technologies, such as membrane bioreactors (MBRs) and advanced oxidation processes (AOPs).
- Insufficient regulatory frameworks mandating AMR-specific treatment or monitoring standards.
- High costs and technical complexity associated with state-of-the-art technologies, making them inaccessible for many LMICs.
- Absence of real-time monitoring systems to evaluate the effectiveness of HWWT in mitigating AMR.
- Limited awareness among stakeholders about the environmental role of HWW in AMR proliferation.

Experimental approaches in some LMICs have shown promise. In Vietnam, for instance, a combined sponge-Membrane Bioreactor (sponge-MBR) and ozonation system significantly improved antibiotic removal from hospital wastewater (17). This treatment employed physical retention, biodegradation, sorption, and photo-transformation to enhance antibiotic elimination. Despite its potential, however, large-scale implementation is hindered by high costs and the need for comprehensive cost-effectiveness assessments.

In Ethiopia, hospitals have employed conventional treatment methods like filtration, sedimentation, and biocides to manage bacterial contamination (18). However, these approaches have proven insufficient for effectively eliminating ARB; prolonged retention times may even facilitate horizontal gene transfer and resistance development. Similar outcomes have been reported in other LMICs, where treatment methods focused on basic filtration and sedimentation fail to adequately address AMR, allowing resistant bacteria and genes to persist in treated effluent (17–26).

More advanced technologies, such as membrane bioreactors, ozonation, and UV-based Advanced Oxidation Processes (AOPs), have shown success in reducing ARGs and antibiotic concentrations in experimental setups in LMICs (16, 17, 27). Solar-powered UV-AOPs, in particular, have proven effective in eliminating emerging contaminants, making them promising for sustainable AMR mitigation. However, high initial capital and operational costs, technical complexity, and scalability challenges remain significant barriers, limiting their practicality for widespread use in resource-constrained settings. More challenges include,

- Complex maintenance requirements, including frequent cleaning of membranes to prevent fouling.
- Dependence on reliable energy sources, which are often unavailable in resource-constrained settings, although this can be sourced from solar systems.
- Generation of by-products (e.g., reactive oxygen species in AOPs) that may require further treatment.
- Limited local expertise to operate and troubleshoot these advanced systems.

In the context of LMICs, hospital wastewater treatment (HWWT) faces significant monitoring gaps and regulatory shortcomings. Effective monitoring of HWWT systems is essential to ensure compliance with environmental standards and control the spread of AMR. However, monitoring in LMICs largely focuses on conventional parameters, such as biochemical oxygen demand (BOD), chemical oxygen demand (COD), total suspended solids (TSS), and nutrient levels (3, 28). Critical gaps remain in monitoring AMR-specific components, including ARGs, pharmaceutical residues, heavy metals (which act as co-selectors for resistance by creating selective pressures in the wastewater environment that favor the survival and proliferation of resistant microorganisms), and multidrug-resistant organisms. These overlooked parameters are critical for evaluating the true effectiveness of HWWT systems in mitigating AMR risks.

## 3 Path forward for effective HWW management in LMICs

**Figure 2:**
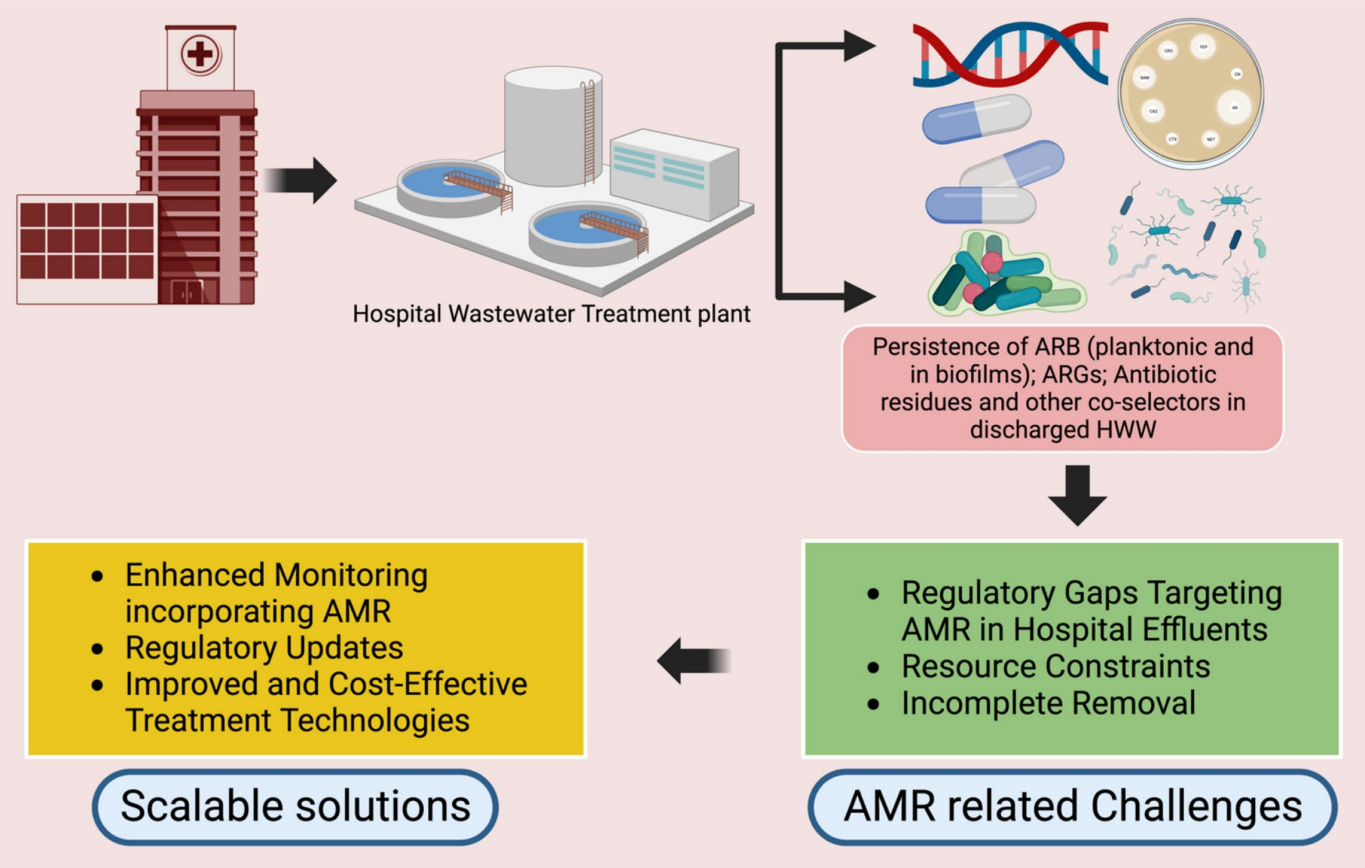
Challenges and perspectives to address the ARG/ARB components in HWWT standards. Created in Biorender.com.

Addressing the current gaps in HWWT requires a multi-faceted approach that includes **expanding onsite treatment**, investing in **innovative technologies**, establishing **standardized monitoring protocols**, and strengthening **collaborative efforts** across sectors (Figure 2). This includes building local technical capacity through training, fostering community engagement, and ensuring financial and policy support for sustainable HWWT systems.

1. **Expanding onsite treatment**: Promoting onsite treatment in hospitals is essential for addressing AMR at its source. By treating wastewater directly at the hospital level, the spread of untreated contaminants into larger ecosystems and urban treatment systems can be mitigated. Modular and affordable decentralized systems can provide practical solutions for LMICs, particularly those with limited infrastructure. Regarding decentralized systems, they could integrate modular components tailored to specific LMIC conditions. For example:
  - A primary sedimentation tank for solids removal.
  - Secondary treatment using constructed wetlands or anaerobic reactors.
  - Tertiary treatment incorporating solar or UV disinfection.
  - Portable, prefabricated units that can be easily deployed in remote or underserved areas.
  - These designs should leverage locally available materials, such as indigenous plants for wetlands, to reduce costs and enhance community acceptance.
2. **Investment in innovative technologies**: Developing cost-effective treatment technologies specifically aimed at reducing AMR is essential for progress. Solutions such as solar-supported UV-Advanced Oxidation Processes (AOPs), sponge-Membrane Bioreactor (sponge-MBR) systems, and other advanced oxidation processes (AOPs) must be tailored to the specific needs and challenges of LMICs, with a focus on minimizing operational complexities. Promising options may include:
  - **Constructed wetlands**: Low-cost, decentralized systems using plants and microorganisms to treat wastewater. Some designs incorporate biofilms to enhance ARG removal.
  - **Solar-driven advanced oxidation processes (AOPs)**: Cost-effective for ARG degradation in sunny regions, though maintenance and scalability remain challenges.
  - **Combined systems**: Sponge-membrane bioreactors coupled with ozonation have shown efficacy in experimental setups in Vietnam.
3. **Adaptation of successful treatment methods**: Treatment methods that have shown promise in other low- and middle-income countries (LMICs) should be adapted for use in different countries or hospitals within the same country. This adaptation process should include in-depth cost-effectiveness and stakeholder analyses to facilitate their successful adoption. So far, several barriers hinder the widespread adoption of successful HWWT methods from one LMIC to another and these may include:
  - Economic disparities, limiting the affordability of advanced treatment technologies.
  - Variability in local infrastructure and technical capacity.
  - Differences in regulatory standards and enforcement mechanisms.

Some relevant solutions could involve:

- Subsidizing technology transfer through international funding initiatives.
- Adapting technologies to local contexts (e.g., using region-specific materials or energy sources).
- Building regional collaborations to share knowledge and experiences.

1. **Tailoring context-specific solutions and leveraging good practices**: Tailored solutions that consider the specific social, economic, and infrastructural contexts of LMICs are essential for effective hospital wastewater management. By integrating local knowledge and building upon existing good practices from similar settings, solutions can be better adapted to meet the unique challenges of each region. This approach not only enhances community engagement but also increases the likelihood of successful implementation and long-term sustainability. Examples may include the use of locally sourced materials for treatment systems and incorporating traditional ecological knowledge into wastewater reuse practices. A few example include systems, such as:
  - Using locally available plants in constructed wetlands to enhance contaminant removal.
  - Incorporating natural coagulants, such as *Moringa oleifera* seeds, for wastewater clarification.
  - Aligning treatment practices with existing community water management systems to foster acceptance and participation.
2. **Standardizing AMR monitoring protocols**: Updated monitoring standards that explicitly include AMR-related parameters, such as ARGs, antibiotic residues, and heavy metals are essential for adequately assessing HWWT performance. Standardized protocols should expand beyond current World Health Organization guidance, which is limited to antibiotic manufacturing wastewater (29), to encompass regular HWWT. This expansion would facilitate better regulation and provide a more comprehensive evaluation of how effectively treatment systems mitigate AMR risks. A robust monitoring protocol should include:
  - Regular quantification of ARGs and ARB using molecular methods (e.g., qPCR or metagenomics).
  - Detection and quantification of antibiotics, disinfectants, and heavy metals.
  - Microbial community analysis to identify resistant species.
  - Standardized sampling points at hospital discharge outlets and downstream ecosystems.
  - Benchmarks for resistance levels, aligned with international guidelines, such as the World Health Organization’s guidance on AMR monitoring in wastewater.
3. **Capacity building and collaboration**: Strengthening local expertise through continuous training initiatives is essential ensuring the sustainability of HWWT solutions. Cross-sector collaboration between healthcare facilities, policymakers, environmental experts, and community organizations, such as the I-CRECT-Consortium,^1^ is vital for advancing the development and adoption of effective AMR mitigation strategies. Such international consortia can:
  - Facilitate knowledge sharing and capacity building among LMIC stakeholders including adoption of HWWT methods that work in one context to another.
  - Support pilot studies and research to evaluate innovative HWWT technologies in diverse contexts.
  - Advocate for harmonized international standards and funding for AMR mitigation efforts in LMICs.
4. **Regulatory reform**: Establishing and enforcing robust regulatory frameworks that mandate AMR monitoring and limit AMR components in treated effluents is essential for meaningful progress. Government support, along with incentives for hospitals that implement effective onsite treatment, could significantly enhance HWW management efforts. Furthermore, governments can:
  - Provide financial subsidies or tax incentives for hospitals that install compliant onsite systems.
  - Establish grant programs for pilot projects in public hospitals.
  - Introduce regulatory requirements with phased implementation to allow hospitals time to comply.
  - Recognize hospitals with effective HWWT systems through public awards to encourage participation.

## 4 Conclusion

Antimicrobial resistance is one of the most pressing public health crises, driven, amongst other factors, by inadequate management of hospital wastewater, particularly in low- and middle-income countries. The discharge of untreated or inadequately treated wastewater, which is rich in antibiotics, ARB, and ARGs, contributes to the proliferation of AMR, adversely affecting both human health and environmental integrity. To effectively combat AMR from an environmental perspective, it is crucial to expand HWW management’s focus beyond conventional pollutants and emphasize resistance control.

This perspective advocates for a transformative shift in HWW management, particularly in LMICs. Key components of an effective strategy include the implementation of onsite treatment, the development of scalable technologies, the establishment of AMR-focused monitoring protocols, and regulatory reform. Collaborative action among researchers, public health officials, environmental regulators, and local stakeholders is imperative for developing innovative solutions, ensuring sustainability, and enforcing compliance with best practices.

Ultimately, and in line with what other researchers in this field have recommended (30), to mandate the monitoring and reduction of AMR components in hospital effluents will require a lot, some of which include:

- Promoting universal implementation of onsite treatment for HWW in hospitals
- Aligning national policies with international guidelines, such as those from WHO.
- Establishing penalties for non-compliance alongside incentives for compliance.
- Integrating AMR-specific parameters into existing water quality standards.
- Ensuring regular audits and transparent reporting of monitoring data.
- Increasing public awareness campaigns about AMR and environmental health connections.

## Footnotes

^1^ https://www.jpiamr.eu/projects/i-crect

## Group members of I-CRECT-Consortium

Linh Viet Nguyen: Hanoi University of Public Health, Hanoi, Vietnam; Tung Xuan Nguyen: Department of Microbiology, Vietnam National Children’s Hospital, Hanoi, Vietnam; Troung Nhat My: Vietnamese German Center for Medical Research (VG-CARE), Hanoi, Vietnam; Lan Thi Le: Department of Microbiology, Vietnam National Children’s Hospital, Hanoi, Vietnam; Huyen Thi Vu: Department of Microbiology, Vietnam National Children’s Hospital, Hanoi, Vietnam; Ngoc Thi Bich Hoang: Department of Microbiology, Vietnam National Children’s Hospital, Hanoi, Vietnam; Dien M. Tran: Director Board, Vietnam National Children’s Hospital, Hanoi, Vietnam; Thi Anh Mai Pham: Department of Global Public Health, Karolinska Institutet, Stockholm, Sweden; Institute of Tropical Medicine, Universitätsklinikum Tübingen, Tübingen, Germany; Vietnamese German Center for Medical Research (VG-CARE), Hanoi, Vietnam; Department of Infectious Diseases and Microbiology, University of Lübeck and University Hospital Schleswig-Holstein, Lübeck, Germany; Phuc D. Pham: Hanoi University of Public Health, Hanoi, Vietnam; Dennis Nurjadi: Institute of Medical Microbiology, University of Lübeck and University Hospital Schleswig-Holstein, Lübeck, Germany; Flavie Goutard: The French Agricultural Research Centre for International Development (CIRAD), Montpellier, France; Thirumalaisamy P. Velavan: Institute of Tropical Medicine, Universitätsklinikum Tübingen, Tübingen, Germany; Faculty of Medicine, Duy Tan University, Da Nang, Vietnam; Van Anh Thi Dinh: Training and Research Institute for Child Health, Vietnam National Children’s Hospital, Hanoi, Vietnam; Department of Infection Control, Vietnam National Children’s Hospital, Hanoi, Vietnam; Bent Jörgensen: Department of Global Studies, Göteborg University, Gothenburg, Sweden; Training and Research Academic Collaboration (TRAC), Sweden, Vietnam; Le Huu Song: Director Board, 108 Military Central Hospital, Hanoi, Vietnam; Nhung T.T. Nguyen: Hanoi University of Public Health, Hanoi, Vietnam; Training and Research Institute for Child Health, Vietnam National Children’s Hospital, Hanoi, Vietnam; Etienne Loire: The French Agricultural Research Centre for International Development (CIRAD), Montpellier, France; Åse Östholm: Department of Biomedical and Clinical Sciences (BKV), Linköping University, Linköping, Sweden; Lennart E. Nilsson: Department of Biomedical and Clinical Sciences (BKV), Linköping University, Linköping, Sweden; Tuyet Hanh T. Tran: Hanoi University of Public Health, Hanoi, Vietnam; Phuc H. Phan: Director Board, Vietnam National Children’s Hospital, Hanoi, Vietnam; Mattias Larsson: Department of Global Public Health, Karolinska Institutet, Stockholm, Sweden; Training and Research Academic Collaboration (TRAC), Sweden, Vietnam; Linus Olson: Department of Global Public Health, Karolinska Institutet, Stockholm, Sweden; Department of Women’s and Children’s Health, Karolinska Institutet, Stockholm, Sweden; Training and Research Academic Collaboration (TRAC), Sweden, Vietnam; Håkan Hanberger: Department of Biomedical and Clinical Sciences (BKV), Linköping University, Linköping, Sweden; Training and Research Academic Collaboration (TRAC), Sweden, Vietnam.
